# Gender and Age Stratified Analyses of Nutrient and Dietary Pattern Associations with Circulating Lipid Levels Identify Novel Gender and Age-Specific Correlations

**DOI:** 10.3390/nu10111760

**Published:** 2018-11-14

**Authors:** Huifeng Jin, Jessie Nicodemus-Johnson

**Affiliations:** Research and Development, USANA Health Sciences, Inc. 3838 W. Parkway Blvd., West Valley City, UT 84120, USA; Huifeng.Jin@us.usana.com

**Keywords:** nutrition, gender, age, dyslipidemia, LDL cholesterol, TG, HDL cholesterol

## Abstract

Dyslipidemia is a precursor to a myriad of cardiovascular diseases in the modern world. Age, gender, and diet are known modifiers of lipid levels, however they are not frequently investigated in subset analyses. Food and nutrient intakes from National Health and Nutrition Examination Study 2001–2013 were used to assess the correlation between lipid levels (high-density lipoprotein (HDL) cholesterol, triglycerides (TG), low-density lipoprotein (LDL) cholesterol, and total cholesterol (TC):HDL cholesterol ratio) and nutritional intake using linear regression. Associations were initially stratified by gender and significant gender correlations were further stratified by age. Analyses were performed at both the dietary pattern and nutrient level. Dietary pattern and fat intake correlations agreed with the literature in direction and did not demonstrate gender or age effects; however, we observed gender and age interactions among other dietary patterns and individual nutrients. These effects were independent of ethnicity, caloric intake, socioeconomic status, and physical activity. Elevated HDL cholesterol levels correlated with increasing vitamin and mineral intake in females of child bearing age but not males or older females (≥65 years). Moreover, increases in magnesium and retinol intake correlated with HDL cholesterol improvement only in females (all age groups) and males (35–64), respectively. Finally, a large amount of gender-specific variation was associated with TG levels. Females demonstrated positive associations with sugar and carbohydrate while males show inverse associations with polyunsaturated fatty acid (PUFA) intake. The female-specific association increased with the ratio of carbohydrate: saturated fatty acid (SFA) intake, suggesting that gender specific dietary habits may underlie the observed TG-nutrient correlations. Our study provides evidence that a subset of previously established nutrient-lipid associations may be gender or age-specific. Such discoveries provide potential new avenues for further research into personalized nutritional approaches to treat dyslipidemia.

## 1. Introduction

Cardiovascular disease (CVD) accounts for more than half of all non-communicable diseases and has become the leading cause of death worldwide [1]. Dyslipidemia, defined as increased triglyceride (TG), low-density lipoprotein (LDL cholesterol) levels, or reduced high-density lipoprotein (HDL cholesterol) levels, is a well-established risk factor for CVD and is estimated to account for more than half of the worldwide cases of coronary artery disease. Diet is a key modifiable lifestyle factor associated with dyslipidemia [2,3,4,5,6,7] and mitigation of chronic disease [8]. In fact, the primary treatment for dyslipidemia is weight loss and diet, specifically reductions in total fat intake [9]. Additionally, intake of soluble fibers, soy isoflavones and nuts have been shown to improve LDL cholesterol levels [10]. While the effects of diet composition on lipid profiles, specifically with respect to fat intake are well understood, the investigation of additional nutrients and dietary patterns in gender or age specific subgroups is less so.

Studies investigating the differences between nutritional intake and relevant phenotypes by gender and age, are essential for providing optimal nutritional guidance. The effect of saturated fatty acid (SFA) intake on lipid levels in men relative to women has been well investigated. A low SFA diet improved serum lipid LDL cholesterol levels more in men than women [11,12,13]. Additional studies on variations in fat ratio intake on lipid levels by gender demonstrated little difference [14]. Studies have also shown that vitamin and mineral intake is correlated with improvements in HDL cholesterol [15,16,17,18]. Individual nutrient levels have also been correlated with lipid profiles [18,19,20,21,22], however these aspects and others are seldom studied with respect to gender or age. A better understanding of the association between lipid levels and nutritional intake by gender and age, above and beyond fat intake, may provide additional avenues for personalized lifestyle approaches to the treatment of dyslipidemia.

Dietary patterns, or the varied combination of nutrients consumed in totality by individuals are a relatively recent approach to nutritional epidemiology that study the combined influence of diet on individual health outcomes, such as lipid levels. In comparison to individual nutrient approaches, this approach is thought to better account for residual confounding of co-consumed dietary components by analyzing the effect of overall diet in lieu of individual nutrients on outcomes of interest (reviewed in [23]). However, this approach is limited by its reduced ability to detect associations of individual nutrients that may not group with complex dietary patterns. Thus, we believe these approaches complement each other and should be used together to fully understand the dietary associations with health outcomes, as observed recently for metabolic syndrome [18].

We examined the correlation between dietary pattern and individual nutrient intake on lipid levels, TG, HDL cholesterol, LDL cholesterol, TC:HDL cholesterol ratio, separately in males and females using data from the National Health and Nutrition Examination Survey (NHANES). Specifically, we investigated the association of age on significant correlations identified in gender analyses using both dietary pattern and nutrient by nutrient analyses. Our integrative approach identified novel dietary patterns and individual nutrients that were correlated with lipid levels in a gender or age-specific manner.

## 2. Materials and Methods

### 2.1. Study Population

The 2001–2013 National Health and Nutrition Examination Study (NHANES) data was used for the analysis. Conducted by the National Center for Health Statistics, NHANES is a stratified, multistage probability sample of the civilian non-institutionalized U.S. population. Adults less than 18 years of age, pregnant women, and those that did not indicate ethnicity or whose ethnicity was labeled as “other” were excluded from this analysis. Individuals that did not have lab, exam, or questionnaire results for HDL cholesterol, TG, LDL cholesterol, and TC or any of the covariate information (physical activity, lipid modifying drug usage, socio economic status) were also excluded. Finally, individuals that did not complete the food frequency questionnaire or had an estimated energy consumption ≤600 or >4800 kcal were removed from further analyses.

### 2.2. Measures

*Nutrition.* NHANES methods for assessing subject nutrition have been described elsewhere [24], briefly dietary intake data were based on 24-h recalls, in which estimates of macronutrient, micronutrient, food group, and energy intake were calculated. The food and beverage consumption of the preceding 24-h period are tabulated. Micro and macronutrient quantities for this study were obtained from the first day dietary interview total nutrient intakes.

*Physiological characteristics and questionnaire information.* All variables were collected from the NHANES database by year and combined using R (version 3.4.1, R Foundation for Statistical Computing, Vienna, Austria). HDL cholesterol, TG, LDL cholesterol, and TC were extracted from the NHANES database as was information on lipid modifying drugs, poverty to income ratio, gender, age, ethnicity, and physical activity. Collection methods have been reported previously [24].

### 2.3. Statistical Analyses

Analyses for NHANES data were conducted according to the guidelines recommended by the Centers for Disease Control, whereby we accounted for masked variance and used the proposed weighting methodology in all analyses [25]. All analyses were performed using R v3.4.1. To better identify dietary predictors of lipid levels that varied by gender, we stratified the analyses by this variable. Because dietary patterns may mask individual nutrient contributions, we performed analyses both on a nutrient by nutrient basis (macro and micronutrients) as well as using dietary patterns. Dietary patterns (of micro/macro nutrient quantities) were derived by principal component analysis (PCA) using the R package survey [26]. For this analysis, nutritional components were first adjusted for total caloric intake using the residuals method. Dietary pattern PCs were binned into quartiles before linear regression analyses. We then regressed lipid levels (outcome) on dietary patterns (predictor variables) while adjusting for age (continuous), race (categorical), poverty to income ratio (less than 1, 1–2, 2–3, ≥3), education (less than high school, high school, more than high school), physical activity (continuous, calculated according to [27]) and use of lipid lowering substances (categorical Yes/No) using linear regression. We also tested quartile associations with linear regression, assuming an additive model, both separately within each gender as well as a gender interaction effect. Linear regression of nutrient level analyses was performed separately in males and females and adjusted for total caloric intake in addition to the covariates listed above for the dietary pattern analysis. As dietary patterns and response to nutrient intake are known to vary by age, we further stratified our datasets by age (18–35, 36–55, >55–65) and re-analyzed significant associations (in at least one gender) to assess the effect of age on our results.

## 3. Results

### 3.1. Population Characteristics

Table 1 shows the population demographics stratified by gender. 12,284 individuals (6127 male and 6157 female) were included in the study. The average age was 46 and 48 for males and females, respectively. The majority of the study population was Caucasian (76 and 75% for males and females respectively). Due to the small subset of non-Caucasian individuals within the study, we adjusted for the effects of ethnicity. As expected, when considering a continuous distribution HDL cholesterol, TG, and TC:HDL cholesterol ratio were significantly different between genders (*p* < 0.001). The observed lipid differences by gender continued when stratified by age. LDL cholesterol levels became significantly different by gender when stratified by age for younger (18–35 year) and older (65–90 year) individuals.

### 3.2. Dietary Pattern/Principal Component Analysis

To identify the main dietary patterns present in our population, we performed PCA on reported macro and micronutrient intakes collectively (Table S1). Three dietary patterns (PCs) each independently explained greater than 5% of the nutritional variance and were used for further analyses. PCs 1–3 explained 10.5%, 9.3%, and 6.5% of the nutritional variation, respectively. Collectively these PCs explained 26.3% of the total variance (Table S1). Because gender may alter the dietary patterns produced in our analyses, we also performed PCA separately in each gender. Comparison of the PC loadings from the combined analysis and gender subset analyses were highly correlated (*p* < 2.2 × 10^−16^ for all PCs; data not shown), thus PCs generated from the combined analysis were used for further studies. Nutrients with an absolute loading value greater than 0.15 were considered to contribute significantly to the dietary pattern. PC1 was negatively correlated with vitamin, mineral, fiber and protein consumption, termed vitamin and mineral intake. PC2 was negatively correlated with folate, carbohydrate, and fiber intake while positively correlated with monounsaturated fatty acid (MUFA) and saturated fatty acid (SFA) intake, termed SFA:carbohydrate. This PC was associated with high saturated and monounsaturated fat and low carbohydrate/fiber intake. PC3 was negatively correlated with SFA, carbohydrate, sugar and calcium consumption while positively correlated with MUFA/polyunsaturated fatty acid (PUFA) consumption, i.e., low carbohydrate and SFA high MUFA/PUFA intake, termed SFA:PUFA. To increase interpretability of dietary pattern analyses we grouped the PCs into quartiles. The mean nutrient intake for each PC quartile stratified by gender is shown in Table S2. The proportion of change was similar between genders for significantly associated nutrients. The average reduction in vitamin, mineral, fiber, and protein (PC1) intake was 50 (males) and 48% (females) between the highest quartile (Q1) and the lowest quartile (Q4). For PC2, the average reduction in folate, fiber and carbohydrates was 41% for males and females, respectively, while the average increase in SFA and MUFA was 1.58 and 1.66 fold for males and females respectively. For PC3, the average reduction in SFAs and sugars was 53 (males) and 50% (females) from the highest quartile (Q1) to the lowest (Q4). The average increase in PUFA and MUFA was 1.77 and 2.25 fold. A large proportion of this increase was attributable to PUFA22:5, docosapentaenoic acid which increased 7.5 and 13 fold in both males and females respectively.

### 3.3. Dietary Pattern Associations with Lipid Levels

**Figure 1:**
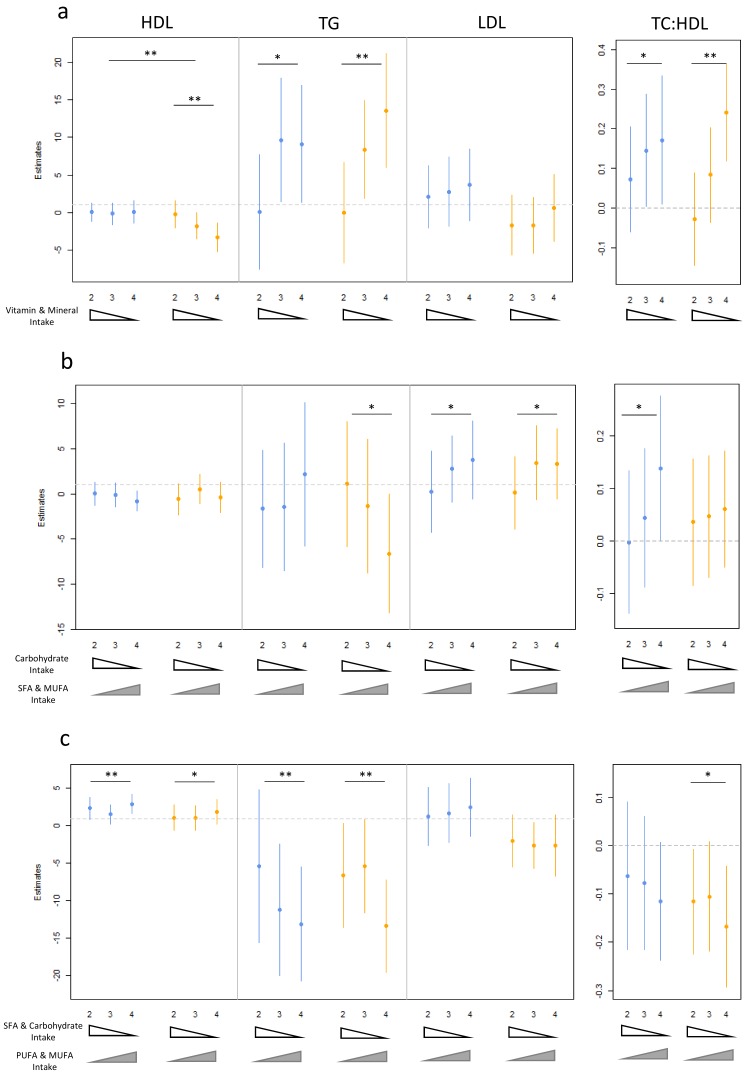
Gender stratified multivariable-adjusted linear association plots (beta co-efficient, 95% confidence interval) of correlation between lipid levels (high density lipoprotein (HDL cholesterol), triglycerides (TG), low density lipoprotein (LDL cholesterol), and total cholesterol to HDL cholesterol ratio (TC:HDL cholesterol)) among dietary pattern quartiles. (**a**) PC1 was correlated with vitamin, mineral, fiber, and protein intake. (**b**) PC2 was correlated with carbohydrate vs. saturated fatty acid (SFA) and monounsaturated fatty acid (MUFA) consumption. (**c**) PC3 was correlated with SFA and carbohydrate vs. polyunsaturated and monounsaturated fatty acid intake. Male and female analyses are shown in blue and orange, respectively. Results are presented as quartile 1 relative to quartiles 2–4 (Q2–4). Triangles below each panel represent the direction of increasing nutrient intake. For example, vitamin and mineral consumption A decreases with each quartile. Double asterisks represent significant associations (*p*_Bonferroni_ < 0.00208) between lipid levels and quartile status assuming an additive model. Single asterisks represent trending associations (0.00208 < *p*_Bonferroni_ < 0.05) under the same model.

We characterized the association of dietary patterns (PC1-3) with lipid levels separately in males and females to better understand the influence of gender. Results were considered significant if they met multiple testing correction of 0.00238 when testing separately in each gender and 0.00714 when testing for an interaction both assuming an additive model (Table S3). Vitamin and mineral intake (PC1) was significantly correlated with three of the four lipids tested (Figure 1A). HDL cholesterol was negatively correlated with PC1 in females (*p*_add_ = 4.30 × 10^−4^) but not males (*p*_add_ = 0.986). This effect was significant for an interaction with gender (*p*_int_ = 1.42 × 10^−6^), and suggests that increasing dietary intake of vitamin and minerals is correlated with reduced risk of low HDL cholesterol levels in females only. TG levels were positively correlated with PC1 in females (*p* = 1.2 × 10^−4^) indicating low vitamin and mineral intake was associated with elevated blood TG. Among males, TG levels were trending toward an association (*p* = 0.00440). No interaction was observed between genders (*p*_int =_ 0.942), suggesting that reduced vitamin and mineral consumption sequentially increased TG levels in both genders albeit to a lesser degree in males. Finally the TC:HDL cholesterol ratio is significantly correlated with vitamin and mineral levels in females (*p* = 1.3 × 10^−4^), and trending in males (*p* = 0.0267). The results in both genders were consistent with reduced vitamin and mineral consumption correlated with risk of elevated TC:HDL cholesterol ratio. We note that although fiber is well known to influence lipid levels [28], removing the effects of fiber via inclusion in our regression model dampens but does not abrogate the association between PC1 and HDL cholesterol (*p*_additive_ = 0.99 (males), 4.3 × 10^−4^ (females)), TG (*p*_additive_ = 0.049 (males), 0.0018 (females)) or TC:HDL cholesterol ratio (*p*_additive_ = 0.23 (males),0.008 (females)), suggesting that fiber does not contribute to the entirety of the observed association.

PC2 (increased SFA: reduced carbohydrate intake) was not significantly associated with lipid levels, however there were many nominal associations (Figure 1B). Increasing female TG levels were modestly correlated with decreasing SFA/MUFA and elevated carbohydrate and fiber consumption (*p*_add_ = 0.029). While this may seem counterintuitive, increasing carbohydrate levels in the presence of reduced SFA are known to increase TG levels [29,30]. Males appear unchanged (*p*_add_ = 0.627). Additionally both male and female LDL cholesterol level increases were nominally associated with low SFA and MUFA and high fiber/folate intake (*p*_add_ = 0.0368 and 0.0396 for males and females respectively), corresponding with the literature demonstrating that increasing carbohydrates while reducing SFA intake is known to increase LDL cholesterol levels [31]. Finally, the TC:HDL cholesterol ratio was positively correlated with PC2 at a nominal *p*-value of 0.0382 in males, while females showed no evidence of an association (*p* = 0.289). The substitution of fatty acids for carbohydrates was beneficially associated with HDL cholesterol metabolism [32], consistent with male results. Although not significant the observed correlative trends were also present in the literature and demonstrate possible interactions with gender for TG and TC:HDL cholesterol ratio that should be further explored.

PC3, high intake of SFA, carbohydrates, sugar and calcium and low intake of MUFA/PUFA were negatively associated with TG levels in males and females, i.e., increased MUFA/PUFA consumption at the expense of SFA intake was associated with reduced TG levels in both genders (*p*_add_ = 1.50 × 10^−4^ and 2.10 × 10^−4^ for males and females respectively). Conversely, HDL cholesterol was positively correlated with PC3 in males (*p*_add_ = 2.5 × 10^−4^) and trends were observed in females (*p*_add_ = 0.0403), suggesting that increased SFA and reduced MUFA/PUFA correlated with reduced risk for low HDL cholesterol in both males and females. An interaction with gender was not detected for either lipid (*p*_int_ = 0.616 and 0.409). TC:HDL cholesterol ratio was trending toward an association with PC3 in females (*p* = 0.0205) and males (*p* = 0.064). The majority of the observed associations with PC3 were observed in both genders suggesting these effects may not vary by gender and demonstrate well documented associations of reduced SFA and increased PUFA intake on lipid profiles [5,7].

We re-analyzed the dataset using a Caucasian only subset and individuals not on lipid modifying drugs only, to assess the influence of ethnicity and lipid modifying medication usage on our results. The same trends were observed in both the full and subset analyses (Table S4). Collectively dietary pattern analyses support the literature demonstrating the association of common dietary patterns with lipid fluctuation while identifying previously unknown gender interactions that may explain variability in reported associations as well as help to increase our understanding of nutritional association with lipid variation in the U.S. population.

### 3.4. Age, Gender and Dietary Pattern Associations with Lipid Levels

Age is known to alter the absorption or subsequent processing of nutritional components, therefore to investigate the effect of age on significant gender-associations we repeated our previous linear regression analysis under an additive model (Table 2). As TG levels are strongly associated with both genders in PCs 1 and 3 these analyses were combined. The association of HDL cholesterol with vitamin and mineral intake (PC1) in females was significant in the young (*p* = 1.94 × 10^−4^; *β* = −1.93 mmol/L) to middle (*p* = 0.027; *β* = −0.95 mmol/L) age demographic with no association in older individuals (>65; *p* = 0.817; *β* = −0.16 mmol/L). Indicating that physiological differences between men and women of child bearing age may interact with vitamin and mineral consumption to modulate the risk of low HDL cholesterol in women. Improvement of TG level correlation with increased vitamin and mineral consumption was observed in all age groups (*p* < 0.05). Although a similar trend was observed in males and females for TC:HDL cholesterol ratio, i.e., improvement was associated with increased vitamin and mineral consumption, the age association results are different. Males show a modest association overall, however associations appear strongest in young and older individuals. The association in females was much stronger in younger individuals and tapers off with increasing age (*p* = 3.26 × 10^−4^ vs. *p* = 0.42; Table 2).

The modest improvement (reduction) in HDL cholesterol levels with increasing MUFA/PUFA and decreased SFA intake (PC3) in males was significant or trending in all age groups. TG trends with PC3 were also detected in all age groups (*p* < 0.1; Table 2). These analyses suggest that in addition to minimal gender alterations, PC3 associations may not be affected by age. Collectively, these age analyses build on the gender effects observed previously, and add information on age associations that are necessary for study interpretation.

### 3.5. Individual Nutrient Consumption Associations with Lipid Levels

**Figure 2:**
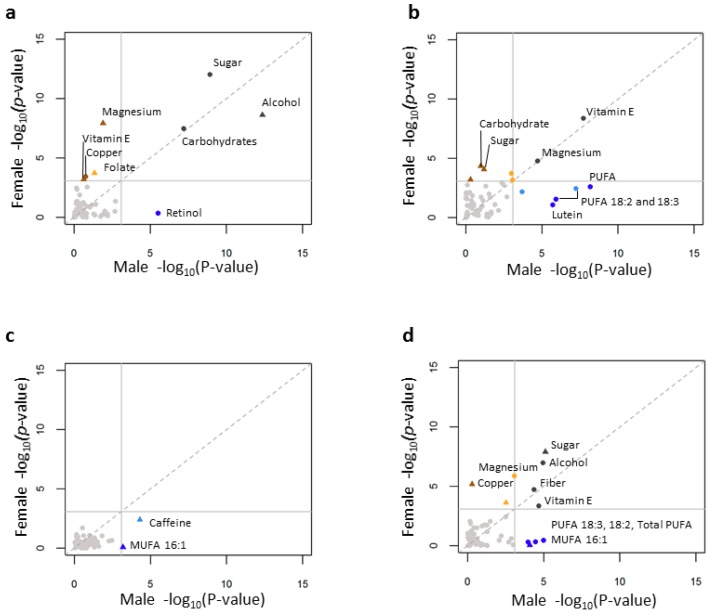
Scatter plot showing the correlation between multivariable-adjusted linear association of circulating lipid levels and individual nutrients in males and females. The x and y axes represents log_10_ (*p*-values) for female and male analyses of HDL cholesterol (**a**), TG (**b**), LDL cholesterol (**c**), and TC:HDL cholesterol ratio (**d**). The Bonferroni adjusted *p*-value cutoff (*p* = 0.000833) is indicated as the horizontal and vertical solid gray line. Non-significant points are shown in grey. Grey points correspond to significant associations in both genders. Significant female or male only points are shown in brown and orange or blue, respectively. Brown and dark blue points represent sites where the beta-coefficient of the significant association is > 2× that of the non-significant association. Orange and light blue represent a 1.5× difference.

In an effort to expand on dietary pattern analyses and identify individual nutrients that may influence lipid levels (*p* < 0.000833; Figure 2; Table S5) we analyzed the dietary intake data on a nutrient by nutrient basis. Carbohydrate and sugar intake were negatively correlated while alcohol intake was positively correlated with HDL cholesterol levels in both genders (Figure 2A; Table S5), consistent with the literature [33,34]. Magnesium intake was positively associated with HDL cholesterol levels in females only. We did not observe an association with age (Table S6), suggesting this association may be independent from the dietary pattern analysis. Additional female-specific associations (Vitamin E and Copper) were also observed. Copper was previously shown to be negatively associated with HDL cholesterol levels in children [35] and among a predominantly female population of adults [36]. Vitamin E was associated with reduced HDL cholesterol levels in animal [21,37] and adult human [38] studies. Finally, we also report a male-specific effect of retinol intake, such that reduced intake of retinol was associated with increased HDL cholesterol. Note that while these associations were statistically significant and novel the effect was modest making detection in gender-corrected studies less likely.

TG levels were negatively correlated with vitamin E and magnesium intake in both genders, consistent with the literature [39,40] (reviewed in [41]). Among males, lutein and PUFA intake were negatively correlated with TG (Figure 2B; Table S5). The intake of good fats (PUFAs) are known to reduce TG levels [7,8], consistent with this observation in males. However, PUFA intake was not correlated with TG levels in females. Additionally, carbohydrate and sugar intake were positively correlated with TG levels in females only. This female-specific correlation of increased carbohydrate/sugar intake with TG levels was consistent with dietary pattern analysis in which PC2 SFA vs. carbohydrate intake was nominally associated with TG levels in females but not males in the same manner (Figure 1B). Moreover, females have a much lower intake of SFA than males (*p* < 2.2 × 10^−16^, *β* = −7.3). High carbohydrate:SFA ratio was associated with increased TG levels [5]. To this end we tested the association of TG with carbohydrate intake by quartiles of carbohydrate:SFA ratio, to test if the correlation of carbohydrates with TG increases with increasing SFA:carbohydrate ratio. As expected, among females but not males, we observed that the correlation between TG and carbohydrate increased as the ratio of carbohydrates to SFA increased (Table S7), highlighting nutritional/behavioral differences between genders that result in different nutrient phenotype correlations.

There were few individual nutrient interactions with LDL cholesterol. Caffeine and MUFA16:1 were positively associated with LDL cholesterol levels (*p* = 5.02 × 10^−5^) (Figure 2C; Table S5). The ratio of total cholesterol to HDL cholesterol was positively correlated with sugar and negatively correlated with alcohol, fiber, and vitamin E in both genders (Table S5). Magnesium was negatively while copper was positively correlated with TC:HDL cholesterol in females only. Among males PUFAs (Total PUFA, PUFA 18:2, and PUFA 18:3) were negatively correlated while MUFA 16:1 was positively correlated with TC:HDL cholesterol ratio, consistent with the literature where increased PUFA intake reduces TC levels and raised HDL cholesterol levels [32]. The gender associations in the ratio likely reflect the gender associations observed in HDL cholesterol and TG. As TC to HDL cholesterol ratio is considered to be a very good predictor of specific types of cardiovascular disease risk [5] further understanding of nutritional and gender interactions is warranted. Furthermore, an understanding of how the different components that make up the phenotypes (i.e., TC:HDL cholesterol ratio involves LDL cholesterol, TG, and HDL cholesterol) are involved with the outcome of interest can increase interpretation of relevant phenotypes and further promote understanding of potential nutritional intervention and treatment.

### 3.6. Age, Gender and Individual Nutrient Associations with Lipid Levels

To test if significant nutrient level correlations with lipid levels varied by age, we subset the individual nutrient data by gender and age groups (18–35, 36–64, ≥65) and repeated the linear regression analyses (Table S6). Among males, HDL cholesterol levels were correlated with carbohydrates, sugar, and alcohol consumption in all age groups, however alcohol consumption showed an interaction with age such that increased alcohol intake was associated with a larger improvement in HDL cholesterol levels as males aged (*p*_int_ = 0.00136). The entirety of the male-specific retinol association with HDL cholesterol levels was observed in the 36–64 year age group (*p* = 2.87 × 10^−8^; *p* = 0.82 and 0.35 for ≤35 and ≥65 age groups respectively). Among females HDL cholesterol levels were associated with folate and alcohol in an age specific manner. Associations with HDL cholesterol levels and alcohol consumption increased with age while the correlation with folate intake and HDL cholesterol levels decreased with age.

Among males, TG levels were correlated with significant individual nutrient intake in all age groups. Vitamin E levels trended toward an interaction (*p* < 0.05) with age such that increasing age displayed a more prominent improvement in TG levels. This effect was not observed to the same degree in females (*p*_int_ = 0.72). Female-specific carbohydrate and sugar associations were correlated with age such that older females demonstrated a larger inverse association with TG levels (*p*_int_ = 0.0084 and 0.03, respectively). TC:HDL cholesterol ratio showed many age associations. Sugar demonstrated a positive association with TC:HDL cholesterol levels and this association strengthened with age in males (*p*_int_ = 6.68 × 10^−3^). The negative correlation of PUFA intake with TC:HDL cholesterol ratio also showed evidence of age associations in males (*p*_int_ = 0.032). Magnesium, copper, and fiber intake were negatively correlated with TC:HDL cholesterol ratio in females. This association also decreased with age (Table S6).

## 4. Discussion

Overall our dietary pattern results are consistent between genders and the literature. However, as observational datasets become larger and we have the capability to stratify data and perform subset analyses to tease apart subphenotype or subpopulation effects we may find novel nutritional associations within subpopulations that expand the capabilities of personalized nutrition. For example, HDL cholesterol was shown to improve with increasing intakes of many vitamin and minerals in mixed gender interventions [16,17], and a cross-sectional study of younger (<55 years) obese Chinese women [15]. Although consistent in direction with the present findings, in taking into account gender and age we report a novel gender and age specific association, warranting further validation of this gender and age specific effect in intervention studies. One mechanism for this female specific effect may be tied to the association of vitamin and minerals to estrogen processing [42,43,44,45,46] and the subsequent link to estrogen-mediated HDL cholesterol production via *ApoA1* [47,48]. This effect is not observed in older women (>65) likely due to postmenopausal drop in estrogen or the high prevalence of hormone replacement therapies. Although other lipid modifying components such as fiber are included in this association, we believe the majority of this effect in HDL cholesterol and TG may be due to co-consumed vitamin and minerals as fiber has been shown to have limited effects on HDL cholesterol and TG levels [28] (reviewed in [49]). In fact fiber is known to reduce improvements in pre- relative to postmenopausal women [50] while the inverse is observed in our dataset.

In addition to the interaction observed in the dietary pattern analyses, many gender-specific associations were observed in nutrient by nutrient analyses. These associations, if reported in the literature are reported for mixed gender studies. For example, we identified magnesium and folate intake as female-specific associations with low HDL cholesterol levels. Although these minerals have been associated with HDL cholesterol levels previously [18,22] we present the first instance of a gender-specific effect. Additionally, the male-specific association of retinol with HDL cholesterol is in agreement with the literature in which retinol consumption has been shown to lower HDL cholesterol in a large 3 year mixed gender intervention trial [19], however our data suggests that males between 36 and 64 may only exhibit this effect. This further suggests that the influence of these nutrients on lipid levels may vary significantly by gender and age and should be further explored in future intervention studies. In addition to mixed gender studies that show similar effects, we identify gender-specific nutritional associations that are conflicting in the literature, such as copper. Low copper levels have been associated with hypercholesterolemia [20,51], however the data is inconsistent and the presently reported gender-specific associations may help explain this observation. These analyses demonstrate the importance of data stratification to better understand nutritional associations in population subsets and may increase the potential to improve health through personalized nutrition.

As the nutrition field shifts from nutrient by nutrient to dietary pattern analyses, our studies highlight the fact that these analyses are not mutually exclusive but complementary to one another in understanding associations as well as highlighting both overall pattern and individual nutrient effects. For example, males show no effect with dietary pattern and HDL cholesterol, while the nutrient by nutrient analyses show common associations with HDL cholesterol such as sugar and alcohol, both of which have been linked to lipid metabolism and clearance [52,53] (reviewed in ref. [54]). Similarly, triglyceride levels were associated with vitamin and mineral (PC1) as well as PUFA vs. SFA (PC3) dietary patterns, however nutrient by nutrient analyses add to this by identifying magnesium and vitamin E intake as prominent contributors. Additionally, the female-specific effects of sugar and carbohydrates reached significance at the nutrient level (as opposed to a trend in dietary pattern analysis; PC2) likely because the dietary pattern association is diluted by co-consumed nutrients. Moreover, the gender dichotomy between nutrient intake and TG levels is more apparent and more clearly due to gender-specific dietary differences (i.e., increased carbohydrate (females) and increased SFA (males) consumption) as opposed to physiological differences. This is consistent with literature demonstrating that environment in which SFA is consumed contributes substantially to SFA effect on lipid levels [8]. Overall, these data demonstrate how dietary pattern and nutrient by nutrient analyses together may facilitate increased interpretation of nutrition data.

We note limitations to our study. Being a cross-sectional study, our results cannot infer causality; only demonstrate an association that must be validated in further nutritional or dietary intervention trials. Additionally, many genetic variants are known to alter lipid levels (reviewed in [55,56,57]). We cannot rule out the possibility that differences between our study and previously published studies is due to genotypic variation between population or that genetic influences obscure dietary associations [58]. Finally, there are few nutritional associations reported for LDL cholesterol. Many LDL cholesterol phenotype related associations are driven by particle size (reviewed in [59]), while our analysis encompassed LDL cholesterol levels as whole investigation by particle size was outside the scope of this study.

## 5. Conclusions

Overall, our study identified multiple novel gender and age specific nutritional associations within the US population, providing evidence that a subset of previously established nutrient-lipid associations may be gender or age-specific. Such discoveries provide potential new avenues for further research into personalized nutritional approaches to treat dyslipidemia.

## Figures and Tables

**Table 1:** Distribution of population demographics and lipid profiles by gender.

	Male	Female	Mean Difference	*p*-Value
**Participants (*N*)**	6127	6157	-	-
**Estimated Population**	34,634,163	36,631,640	-	-
**Age (mean ± SE)**	45.93 ± 0.36	47.61 ± 0.33	-	-
**Race (proportion)**				
Non-Hispanic White	0.76	0.75	-	-
Non-Hispanic Black	0.1	0.12	-	-
Mexican American	0.087	0.078	-	-
Other Hispanic	0.05	0.05	-	-
**HDL cholesterol(mg/dL) (mean ± SE)**	48.61 ± 0.24	59.08 ± 0.33	−10.47	<0.001
18–35	47.64 ± 0.36	55.93 ± 0.50	−8.29	<0.001
36–64	48.65 ± 0.38	59.80 ± 0.47	−11.15	<0.001
65–90	50.31 ± 0.58	61.75 ± 0.58	−11.44	<0.001
**Triglycerides (mg/dL) (mean ± SE)**	129.47 ± 1.53	116.57 ± 1.25	12.9	<0.001
18–35	115.35 ± 2.52	97.96 ± 1.91	17.39	<0.001
36–64	138.0 ± 2.08	119.56 ± 1.74	18.44	<0.001
65–90	129.92 ± 2.19	135.95 ± 2.59	−6.03	<0.001
**LDL cholesterol (mg/dL) (mean ± SE)**	116.27 ± 0.67	115.79 ± 0.63	0.48	0.647
18–35	110.25 ± 1.30	102.99 ± 0.95	7.26	<0.001
36–64	123.18 ± 0.82	121.87 ± 0.99	1.31	0.83
65–90	106.47 ± 1.19	118.08 ± 1.20	−11.61	<0.001
**TC:HDL cholesterol ratio (mean ± SE)**	4.15 ± 0.024	3.55 ± 0.020	0.6	<0.001
18–35	4.01 ± 0.046	3.38 ± 0.034	0.63	<0.001
36–64	4.34 ± 0.032	3.65 ± 0.029	0.69	<0.001
65–90	3.84 ± 0.044	3.53 ± 0.036	0.31	<0.001

SE stands for standard error.

**Table 2:** Significant lipid by nutrient correlations stratified by age and gender.

**Vitamin and Mineral Consumption (PC1)**							
		18–35		36–64		65–90	
		*p*_additive_	Estimate (CI)	*p*_additive_	Estimate (CI)	*p*_additive_	Estimate (CI)
HDL cholesterol	Male	0.059	−0.60 (−1.21, 0.015)	0.176	0.54 (−0.23, 1.31)	0.495	−0.39 (−1.42, 0.69)
	Female	1.94 × 10^−4^	−1.93 (−2.90, −0.95)	0.027	−0.95 (−1.79, −0.12)	0.817	−0.16 (−1.49, 1.17)
TG	Combined	3.02× 10^−3^	4.15 (1.48, 6.83)	2.03 × 10^−3^	4.19 (1.60, 6.78)	7.02 × 10^−4^	6.36 (2.80, 9.92)
TC:HDL cholesterol	Male	0.035	0.082 (0.00704, 0.16)	0.445	0.028 (−0.044, 0.10)	0.029	0.091 (0.019, 0.12)
	Female	3.26 × 10^−4^	0.124 (0.059, 0.190)	9.44 × 10^−3^	0.071 (0.019, 0.12)	0.428	0.038 (−0.056, 0.13)
**MUFA/PUFA vs. SFA Intake (PC3)**							
		18–35		36–64		65–90	
		*p*_additive_	Estimate (CI)	*p*_additive_	Estimate (CI)	*p*_additive_	Estimate (CI)
HDL cholesterol	Male	9.03 × 10^−3^	1.00 (0.26, 1.74)	7.19 × 10^−2^	0.52 (−0.04, 1.08)	3.74 × 10^−3^	1.45 (0.60, 2.41)
	Female	0.246	0.45 (−0.31, 1.23)	8.93 × 10^−2^	0.68 (−0.10, 1.45)	0.319	0.48 (−0.46, 1.43)
TG	Combined	5.06 × 10^−3^	−4.24 (−7.13, −1.34)	9.32 × 10^−5^	−4.72 (−6.98, −2.45)	1.29 × 10^−3^	−4.51 (−7.17, −1.84)

## Supplementary Materials

The following are available online at http://www.mdpi.com/2072-6643/10/11/1760/s1, Table S1: Principal component of nutrient intake analysis. PCs displayed explain at least 5% of variance in the data. Loading values greater than 0.15 are bolded, Table S2: Mean of nutrient intake across quartiles of principle components that explain at least 5% of variance in the data, Table S3: Dietary Pattern association with lipid levels stratified by gender. Results are given separately for each quartile relative to Q1, Table S4: Correlation of lipid levels with PC quartiles. The full anaysis is shown as well as analyses subset to Caucasian only and those not taking lipid meds, Table S5: Estimates and *p*-values for significant associations between serum lipid level correlation with individual nutrient intake stratified by gender, Table S6: Significant *p*-value and estimate for gender and age stratified correlative analyses between individual nutrients and serum lipid levels, Table S7: *p*-values and estimates for correlative analysis between carbohyrate and triglyceride level stratified by quartile of SFA:carbohydrate ratio.
